# Risk factors for post-ERCP pancreatitis and assessment of stent exchange intervals in children with chronic pancreatitis: a single-center retrospective study

**DOI:** 10.1186/s12887-026-07225-3

**Published:** 2026-06-24

**Authors:** Guiping Zhao, Yaxin Wang, Guo Zhang, Chuntao Liu, Mengran Zhao, Peng Li, Fujing Lv, Qianyun Lin

**Affiliations:** 1 Department of Gastroenterology, Beijing Friendship Hospital, Capital Medical University, No. 95, Yong’an Road, Xicheng District, Beijing, 100050 China; 2 State Key Laboratory of Digestive Health, Beijing, China; 3 National Clinical Research Center for Digestive Disease, Beijing, China; 4 Beijing Key Laboratory of Early Gastrointestinal Cancer Medicine and Medical Devices, Beijing, China

**Keywords:** Pediatric ERCP, PEP, ERPD, Pancreatic Stent, Stent Exchange Interval

## Abstract

**Background:**

ERPD serves as the first-line endoscopic interventional therapy for pediatric chronic pancreatitis (CP). However, pediatric-specific risk factors for post-endoscopic retrograde cholangiopancreatography pancreatitis (PEP) and the optimal stent exchange intervals remain poorly defined.

**Methods:**

In this single-center retrospective study, pediatric CP patients (≤ 14 years) undergoing ERPD procedures (2015–2024) were enrolled. PEP was diagnosed per ESGE 2020 criteria (new/worsened abdominal pain + amylase/lipase > 3×ULN). Univariate and multivariate logistic regression identified PEP risk factors. Stent exchange intervals (standard interval vs. long interval) were compared for symptoms and acute pancreatitis occurrence.

**Results:**

A cohort of 51 pediatric CP patients underwent 131 successful ERPD procedures. PEP occurred in 23 procedures (17.6%), all classified as mild and managed conservatively without severe complications. Univariate analysis revealed potential associations between PEP and several variables and multivariate analysis identified that pancreaticobiliary maljunction (PBM) (Adjusted OR = 4.861, 95% CI: 1.143–20.716, *P* = 0.032) and small stent diameter (≤ 5 Fr) (Adjusted OR = 3.083, 95% CI: 1.163–9.495, *P* = 0.047) were associated with increased PEP risk. No significant differences were observed in interval symptoms (standard interval: 22.7% vs. long interval: 27.6%, *P =* 0.693) or acute pancreatitis episodes (9.1% vs. 10.3%, *P =* 0.881) between the two groups.

**Conclusions:**

PBM and small stent diameter (≤ 5 Fr) are associated with increased PEP risk, although causality cannot be established. Extending stent exchange intervals beyond 6 months does not increase the risk of symptoms or acute pancreatitis. Individualizing stent replacement schedules to lower procedural burden appears feasible, which requires further validation in prospective studies.

## Introduction

Chronic pancreatitis (CP) in children is a rare but severely debilitating progressive inflammatory disorder. Characterized primarily by abdominal pain and malabsorption, its symptoms are often insidious and recurrent, significantly impacting children’s development [1]. Left untreated, CP can lead to growth retardation, irreversible pancreatic dysfunction, and an increased risk of pancreatic cancer [2]. Current management strategies consist of various supportive and medical interventions, including pancreatic enzyme replacement therapy (PERT) for exocrine pancreatic insufficiency (EPI), pain control and nutritional support. Endoscopic retrograde pancreatic drainage (ERPD) is the first-line endoscopic intervention for pediatric CP complicated with pancreatic duct obstruction [3].

However, despite its established role in adults, ERCP application in children presents distinct challenges. Indications predominantly involve congenital pancreatobiliary anomalies, contrasting with the focus in adults on biliary diseases. Furthermore, the smaller size and delicate nature of pediatric gastrointestinal tracts increase procedural complexity, precluding direct extrapolation of adult protocols. Notably, ERPD is a primary therapeutic modality in children, differing from common adult ERCP practices. Despite this, critical knowledge gaps persist regarding pediatric-specific risk factors for PEP following stent placement [4, 5]. Factors such as patient age, weight, and stent characteristics (length, diameter) remain inadequately investigated [6]. An additional significant clinical dilemma is the optimal interval for stent exchange. The 2018 ESGE Guideline indicates that if a single 10-Fr plastic stent is placed initially with symptom improvement, continuous stent placement for 1 year is recommended without routine exchange [7]. Children often require repeated ERCPs due to underlying anatomical abnormalities, yet current guidelines lack evidence-based recommendations for exchange timing [8]. While the practice of exchanging stents at 6 months is often referenced in pediatric population, its suitability remains incompletely verified [9].

To address these unresolved questions, we conducted a retrospective single-center study. We enrolled pediatric CP patients undergoing ERCP+ERPD at our institution. Using univariate and subsequent multivariate logistic regression analyses, we aimed to identify independent risk factors for PEP in children. Concurrently, through extended follow-up, we sought to determine the optimal stent exchange interval for ERPD. This study aimed to provide evidence to reduce procedure-related morbidity and optimize the management of children with CP requiring pancreatic duct stenting.

## Methods

### Research design

This study employed a single-center retrospective cohort design, enrolling pediatric patients (aged ≤ 14 years) who underwent ERCP+ERPD at the Digestive Endoscopy Center of Beijing Friendship Hospital, Capital Medical University, between January 1, 2015 and April 30, 2024. The study protocol was approved by the institutional Ethics Committee of Beijing Friendship Hospital.

### Inclusion and exclusion criteria

#### Inclusion criteria

1. Pediatric CP patients with ERCP indications aged ≤ 14 years: intractable abdominal pain caused by imaging-confirmed pancreatic duct stricture, dilation, or anatomical anomalies (e.g., pancreas divisum); chronic pancreatitis accompanied by acute episodes.
2. Cases undergoing ERCP+ERPD at the Digestive Endoscopy Center of Beijing Friendship Hospital, Capital Medical University;

#### Exclusion criteria

1. Cases refusing ERCP treatment;
2. Cases with incomplete or inaccessible clinical data.

### Operational procedures and equipment selection

All procedures were performed with the patient in the prone position. Anesthesia was administered either via intravenous general anesthesia (IV GA) or endotracheal intubation general anesthesia (ET GA). The standard wire-guided cannulation technique was employed during the procedure. In cases of cannulation difficulty, precut sphincterotomy or other assisted cannulation techniques were utilized. Endoscopic systems and compatible accessories, including pancreatic stents, dilation bougies, and dilation balloons (manufactured by Olympus Medical Systems Corp., Tokyo, Japan), were selected based on the patient’s age and clinical characteristics. The specific endoscope models used included the JF-240, JF-260 V, and TGF-260 V. Given the unique nature of pediatric ERCP, to minimize complications arising from operator inexperience, all pediatric ERCP procedures at our institution are exclusively performed by two highly experienced chief physicians. Both operators have each performed over 2000 ERCP procedures prior to undertaking these cases. Children in this cohort did not receive routine peri-procedural Non-steroidal anti-inflammatory drugs (NSAIDs) for PEP prophylaxis, given that the optimal agents and dosages for pediatric patients have not been well established in clinical practice. NSAIDs were administered only in a very small number of cases for individualized clinical indications.

### Diagnostic criteria for PEP and CP

According to the 2020 European Society of Gastrointestinal Endoscopy (ESGE) Guideline on adverse events related to ERCP, the diagnostic criteria for PEP are as follows [10]:

1. Clinical Manifestation: New-onset or significantly worsened abdominal pain following ERCP.
2. Biochemical Evidence: Serum amylase or lipase levels exceed three times the upper limit of normal (ULN) at 24 h post-procedure.

The diagnosis of CP was established based on a comprehensive assessment of clinical manifestations, laboratory examinations, and imaging findings, in accordance with the internationally accepted INSPPIRE criteria [11].

### Observation parameters

1. Patient Demographics and Baseline Characteristics: Age, Sex/Gender, Body weight, Medical history (including relevant comorbidities).
2. Preoperative Laboratory and Imaging Findings: Preoperative biochemical profiles (e.g., liver function tests, renal function tests, electrolytes), Serum amylase levels, Coagulation function tests (e.g., prothrombin time (PT), activated partial thromboplastin time (aPTT), international normalized ratio (INR)), Preoperative imaging results: Abdominal ultrasound, Magnetic resonance cholangiopancreatography (MRCP), computed tomography (CT), electrocardiogram (ECG) findings.
3. Intraoperative ProceduralVariables: Performance of precut sphincterotomy (Yes/No), Removal ofpre-existing pancreatic duct (PD) stent (Yes/No), Number of pancreatic ductstents placed, Length of pancreatic duct stent(s) (mm or Fr), Diameter ofpancreatic duct stent(s) (Fr), Performance of balloon dilation.
4. Postoperative Clinical Symptoms: Presence and severity of: Abdominal pain, Fever, Jaundice, Nausea and/or vomiting, Other procedure-related symptoms.
5. Postoperative Laboratory Parameters: Serum testing within 24 h post-ERCP including: Complete blood count (CBC) with differential + C-reactive protein (CRP), Comprehensive metabolic panel (electrolytes, renal/liver function), Serum amylase and/or lipase.
6. Follow-up Protocol for Pediatric Cohort: Scheduled pancreatic duct (PD) stent exchange intervals. Stent replacement timing was categorized based on the actual duration observed in clinical practice. While the clinical recommendation is for exchange within 6 months, the actual timing varied due to factors such as patient compliance, vacation schedules, and logistical difficulties for patients residing in remote locations. This variation reflects the realities of clinical practice, not a predefined intervention. Thus, we retrospectively defined stents removed within or at 6 months as the standard interval group and those removed after 6 months as the long interval group. Stent-related symptoms during follow-up periods, Incidence of acute pancreatitis (diagnosed per Revised Atlanta Criteria). The symptoms assessed during the follow-up period included persistent or worsening abdominal pain, nausea/vomiting, fever, and jaundice. The follow-up schedule was every 3 months after first stent placement, and then every 6 months thereafter. At each follow-up, patients or their guardians were specifically asked about the presence of the symptoms since the last visit.

### Statistical analysis

Statistical analysis was performed using SPSS 26. Normally distributed data were analyzed using the independent t-test, while non-normally distributed data were analyzed using nonparametric tests (Mann-Whitney U test). Categorical variables were analyzed using the chi-square test, and ranked/ordinal data were analyzed using the Kolmogorov-Smirnov two-sample test. Factors influencing clinical outcomes were analyzed using binary logistic regression models. The statistical significance level was set at *P* < 0.05.

## Results

### Clinical characteristics of the participants

A total of 51 pediatric patients who underwent ERCP+ERPD at our institution were enrolled. The cohort comprised 27 males (52.9%) and 24 females (47.1%), with a marginal predominance of male patients. Baseline data are presented in Table 1.

**Table 1:** Baseline characteristics of the 51 enrolled patients

Characteristic	*n* (%) / Mean ± SD
Gender	*n* (%)
Male	27 (52.9%)
Female	24 (47.1%)
Age (years)	
0–6	6 (11.8%)
7–11	28 (54.9%)
12–14	17 (33.3%)
Height (cm)	141.8 ± 17.5
Weight (kg)	37.5 ± 14.3
BMI(kg/m²)	18.1 ± 3.5
Comorbidity	
Fatty liver	1
Splenomegaly	1

### Endoscopic procedures and stent characteristics

A total of 131 ERCP procedures with pancreatic duct stenting were successfully performed in 51 pediatric patients with CP with a technical success rate of 100%. Nineteen patients (37.3%) underwent single stenting procedures, while 32 patients (62.7%) required two or more procedures. The main endoscopic findings included pancreatic duct strictures (92.3%), pancreas divisum (71.8%), pancreaticobiliary maljunction (12.2%), pancreatic duct stones (6.1%), pancreatic pseudocysts (1.5%) and pancreatic duct fistula (0.7%). Additional intraoperative interventions consisted of precut sphincterotomy (10.7%), stent removal (35.1%), balloon dilation (6.1%), bougienage dilation (15.3%) and pancreatic stone extraction (6.1%). The primary goal of all procedures was pancreatic duct drainage to relieve obstruction, rather than stone removal or post-ERCP pancreatitis prevention.Stents were classified by length, diameter and quantity. We categorized stents longer than 5 cm as the long stent group, and those of 5 cm as the short stent group. Similarly, placement of pancreatic duct stents with diameters greater than 5 Fr was defined as the large caliber stent group, and placement of stents where all were 5 Fr in diameter as the small caliber stent group. The long stent group comprised 108 procedures (82.4%), significantly higher than the short stent group’s 23 procedures (17.6%). The large caliber stent group included 66 procedures (50.4%), while the small caliber stent group included 65 procedures (49.6%), indicating similar procedural volumes between these two groups. One or two stents were placed during each procedure. The single stent group accounted for 74 procedures (56.5%), and the double stent group for 57 procedures (43.5%).

### Risk factors for PEP following pancreatic duct stent placement in children

In 131 ERCP procedures, PEP occurred in 23 cases (17.6%). No other serious complications such as bleeding or perforation occurred. All mild PEP patients were managed in general wards without ICU admission. Their mean postoperative hospital stay was 4.2 days, versus 3.3 days in the non-PEP group. No readmission for recurrent pancreatitis or related complications occurred during follow-up.

We first performed univariate logistic regression analyses to identify risk factors for PEP following ERCP in children, incorporating all previously collected variables (Table 2). The regression analysis revealed that Pancreaticobiliary Maljunction(PBM) (OR: 5.220, 95% CI: 1.015–26.850, *P* = 0.025) and small stent diameter (≤ 5 Fr) (OR: 3.317, 95% CI: 1.033–10.649, *P* = 0.035) may be independent risk factors for PEP in children undergoing ERPD. No other factors were identified as independent risk factors for PEP in children undergoing ERPD.

**Table 2:** Univariate Analysis of Risk Factors for Post-ERPD Pancreatitis

Related factors	Number of cases/total number of cases Incidence rate (%)	Univariate *P* value	Crude OR (95% CI)
Gender		0.128	0.470(0.162–1.361)
Male	11/81(13.6)		
Female	12/50(24.0)		
Age (years)		0.982	1.248(0.312–4.998)
0–11	4/23(17.4)		
12–14	19/108(17.6)		
Weight	7/36(19.4)	0.727	1.517(0.470–4.895)
First ERCP		0.491	1.03(0.341–3.064)
First-time ERCP	5/28(17.9)		
subsequent ERCP	18/103(17.4)		
Acute pancreatitis history	14/84(16.7)	0.720	0.770(0.238–2.497)
Diagnosis			
Complete Pancreas divisum	4/32(12.5)	0.387	0.737(0.184–2.952)
Incomplete Pancreas divisum	11/62(17.7)	0.958	1.219(0.383–3.884)
Pancreaticobiliary maljunction	6/16(37.5)	0.025	5.220(1.015–26.850)
Endoscopic Treatment			
Precut sphincterotomy	2/14(14.3)	0.734	0.471(0.064–3.449)
Pancreatic stent removal	6/46(13.0)	0.318	0.715(0.194–2.633)
Balloon dilation	1/8(12.5)	0.698	0.656(0.057–7.548)
Bougienage dilation	4/19(21.1)	0.665	1.153(0.280–4.748)
Pancreatic stone extraction	2/8(25.0)	0.568	3.119(0.359–27.111)
Stent length		0.562	1.241(0.278–5.547)
Long-stent group	18/108(16.7)		
Short-stent group	5/23(21.7)		
Stent diameter		0.035	3.317(1.033–10.649)
Large-diameter	7/66(10.6)		
Small-diameter	16/65(24.6)		
Stent number		0.352	1.063(0.314–3.595)
Single stent	15/74(20.3)		
Multiple stents	8/57(14.0)		

To exclude confounding factors and increase the statistical power of this analysis, four variables were further included in the multivariate logistic regression model: the two significant factors from univariate analysis (PBM and small stent diameter), plus two widely reported classic risk factors (precut sphincterotomy and first-time ERCP).The results of multivariate logistic regression analysis (Table 3) showed that after adjusting for potential confounding factors, only PBM (Adjusted OR = 4.861, 95% CI: 1.143–20.716, *P* = 0.032) and small stent diameter (≤ 5 Fr) (Adjusted OR = 3.083, 95% CI: 1.163–9.495, *P* = 0.047) were identified as independent risk factors for PEP in children undergoing ERPD (*P* < 0.05). In contrast, precut sphincterotomy and first-time ERCP, which were included as classic risk factors, showed no statistically significant association with PEP in the multivariate analysis (*P* > 0.05).

**Table 3:** Multivariate Analysis of Risk Factors for Post-ERPD Pancreatitis

Related factors	Number of cases/total number of cases Incidence rate (%)	Multivariate *P* value	Adjusted OR (95% CI)
Pancreaticobiliary maljunction		0.032	4.861(1.143–20.716)
Yes	6/16(37.5)		
No	17/115(14.7)		
Precut sphincterotomy		0.894	0.942(0.208–4.261)
Yes	2/14(14.3)		
No	21/117(17.9)		
Stent diameter		0.047	3.083(1.163–9.495)
Large-diameter	7/66(10.6)		
Small-diameter	16/65(24.6)		
First-time ERCP		0.946	0.965(0.293–3.187)
First-time ERCP	5/28(17.9)		
Subsequent ERCP	18/103(17.4)		

We would like to point out that factors such as age, weight, pancreas divisum, prior pancreatitis and pancreatic stone extraction may also carry risks. We further conducted a multivariate analysis including all the above variables and compiled the corresponding tables. The core conclusions derived from the two analytical models were consistent.

### Symptom and acute pancreatitis occurrence by stent exchange interval

This study followed 51 patients postoperatively, stratified into a standard interval group (stent exchange ≤ 6 months, *n* = 22) and long-interval group (> 6 months, *n* = 29), with median stent indwelling times of 175 days and 336 days, respectively. Baseline clinical characteristics were comparable between the two groups with no statistically significant differences (all *P* > 0.05) (Table 4). The primary endpoint assessed interval symptoms and acute pancreatitis episodes: 5 patients (22.7%) in the standard interval group developed symptoms (1 persistent fever, 4 worsening abdominal pain with nausea/vomiting) versus 8 (27.6%) in the long-interval group (4 abdominal pain, 2 abdominal pain with nausea/vomiting, 2 fever), showing no statistically significant difference (*P* = 0.693); acute pancreatitis occurred in 2 vs. 3 patients, respectively, also without statistical significance (*P* = 0.881) (Table 5).

**Table 4:** Baseline Characteristics of Patients in the Two Stent Exchange Groups

Baseline characteristics	Standard interval group (*n* = 22)	Long-interval group (*n* = 29)	*P*
Age at first symptom (years)	10.3 ± 2.31	10.6 ± 2.71	0.672
Height at first symptom (cm)	142.3 ± 21.2	147.9 ± 14.7	0.268
Weight at first symptom (kg)	34.8(17.0, 89.0)	39.0(24.0,71)	0.061
BMI at first symptom (kg/m²)	17.4 ± 3.6	18.5 ± 2.4	0.199
Gender (Male/Female)	12/10	15/14	0.911
Previous acute pancreatitis (yes/no)	14/8	17/12	0.716
Persistent/worsening abdominal pain (yes/no)	4/18	6/23	0.884
Pancreatic duct stricture (yes/no)	17/5	26/3	0.423

**Table 5:** Comparison of clinical outcomes between the two groups

Acute Pancreatitis Episodes	Standard interval group (*n* = 22)	Long-interval group (*n* = 29)	*P*
Stent indwelling days(median, range)	175(32,188)	336(221,630)	
Procedure-related symptoms	5/22	8/29	0.693
Acute pancreatitis episodes	2/22	3/29	0.881

## Discussion

Current international guidelines recommend ERPD as first-line therapy in pediatric CP with ductal obstruction, yet evidence on risk factors for PEP and optimal stent exchange intervals remains limited. Current practice for pediatric ERCP relies heavily on adult protocols, which may not address pediatric-specific challenges such as congenital pancreatobiliary anomalies and smaller ductal anatomy [12, 13]. Our study identified two independent risk factors for PEP in children: PBM and smaller stent diameter. This will provide important reference for identifying patients at high risk of PEP and for its prevention. Moreover, extending the stent exchange interval beyond 6 months demonstrated no significant increase in symptoms or acute pancreatitis episodes compared to exchanges at ≤ 6 months. This supports strategically prolonging exchange intervals to reduce procedural burden on children and lower healthcare costs in pediatric ERCP practice.

PEP is the most common complication of pediatric ERCP [4, 14]. Many factors may affect the success rate of ERCP and the incidence of PEP such as age, endoscopist experience, and indication variations [15–17]. Focusing on pediatric populations, recent studies have identified consistent procedure‑ and disease‑related PEP risk factors, including pancreatic indications, native papilla, difficult cannulation, pancreatic duct manipulation, high procedural complexity, young age, and pancreatic stent placement [18, 19]. In this study, we exclusively included CP as a single disease entity and this may lead to divergent conclusions regarding risk factors of PEP compared to other studies. Our study confirms PBM as a significant contributor to PEP in CP children. PBM may elevate PEP risk through several mechanisms. First, anatomically, PBM facilitates bile-pancreatic juice reflux, while stent placement further elevates pancreatic ductal pressure [20]. Second, the aberrant anatomy increases technical difficulty during pancreatic duct cannulation and may prolong procedure time and require repeated attempts [21]. Potential mitigation strategies include preoperative MRCP for comprehensive anatomical assessment and double-guidewire technique during the procedure.

Another independent risk factor influencing PEP was pancreatic stent diameter. In our study the proposed mechanism, based on fluid dynamics is that small-diameter stents(≤ 5 Fr) have insufficient drainage efficiency, leading to pancreatic duct hypertension. Another research also demonstrated the superior efficacy of 5Fr pancreatic stents over 3Fr stents in preventing PEP among high-risk patients, indicating stent diameter a more critical determinant of PEP prevention than stent length or number of stents [22]. However, a secondary analysis of randomized trial data indicates no significant association between the efficacy of prophylactic pancreatic stent placement in preventing PEP and five modifiable technical factors including stent diameter [23]. The discrepancy between our findings and this study may be attributed to: (i) distinct study population characteristics (age distribution and disease spectrum), and (ii) differences in stent placement aims (therapeutic drainage in our cohort versus pancreatitis prophylaxis in their protocol). Notably, Small-caliber stents (≤ 5 Fr) are commonly applied in younger children and patients with narrow pancreatic ducts. Accordingly, stent size may merely act as a surrogate marker for pancreatic duct anatomy, instead of a direct causal factor for PEP. Therefore, small-caliber stents were associated with increased PEP risk, although causality cannot be established. Considering the main purpose of therapeutic drainage in pediatric patients rather than pancreatitis prophylaxis, larger-diameter stents should be considered when clinically appropriate.

Other studies indicate additional PEP risk factors in children include ERCP procedural (difficult cannulation), intraoperative guidewire insertion into pancreatic duct and pancreatic disease(compared to biliary indication) [18, 19, 24, 25]. However, we exclusively included CP patients and achieved technical success in all cases, association between procedural challenges, guidewire misplacement into pancreatic duct, pancreatic indications and PEP could not be validated in our study. Deep guidewire insertion into pancreatic duct may trigger pancreatitis. Due to the retrospective nature of our study, accurate intraoperative data on cannulation depth/duration were unavailable, precluding statistical analysis of this variable. In the present study, commonly recognized PEP high-risk factors (precut sphincterotomy and first-time ERCP) showed no statistical significance, inconsistent with the established consensus [26, 27]. We speculate this discrepancy may arise from three main reasons: most prior PEP studies focused on biliary procedures (vs. our pancreatic duct interventions), all our patients received pancreatic duct stenting after precut (unlike routine practice), and our study included children under 14 (vs. previous adult-focused studies), with age-related differences in pancreatobiliary physiology potentially altering PEP risk factors. Our findings also highlight a unique risk profile for PEP in the pediatric population. We observed a higher prevalence of PEP (17.6%) in our cohort when compared to some adult studies. This is likely not simply determined by age. Instead, this increased risk appears to be multifactorial, stemming from the inherent anatomical challenges and procedural complexities of treating children with chronic pancreatitis.

Current evidence regarding prophylaxis for PEP in pediatric populations remains limited. Mark JA suggested that NSAIDs such as ketorolac may provide prophylactic value against pediatric PEP. In a randomized controlled trial by Troendle et al., intravenous ibuprofen reduced postprocedural abdominal pain risk (*P* = 0.01) [28]. However, its prophylactic effect against PEP did not reach statistically significant differences (7% vs. 17%, *P* = 0.42) [29]. ESGE recommends routine rectal administration of 100 mg of diclofenac or indomethacin immediately before or after ERCP in all patients without contraindication. However, this guideline primarily focuses on the adult population, and no consensus exists on the optimal NSAID selection or dosing regimens specific for children.

A key finding of this study is that pancreatic stent exchange intervals exceeding six months demonstrated no significant difference in the incidence of procedure-related symptoms or pancreatitis during follow-up, compared with exchanges at ≤ 6 months. ESGE suggests stents should be exchanged if necessary, based on symptoms or signs of stent dysfunction at regular pancreas imaging at least every 6 months [7]. Current controversies exist between scheduled exchange (typically every 3–6 months) and symptom-driven exchange in children, but most studies supporting symptom-driven exchange are now considerably dated [30, 31]. Despite the absence of pediatric guideline recommendations, stent replacement every 6 months is typically recommended in pediatric practice [32, 33]. Our finding supports judiciously extending pediatric stent exchange intervals, contradicting current tacit rules. Crucially, stent exchange should not be indiscriminately prolonged in case of stent dysfunction. Considering the elevated risks of stent occlusion with small-diameter stent for a prolonged period, the higher risk of PEP as mentioned above in small-diameter group, a strategic shift toward large-diameter stent (≥ 6 Fr) with judiciously prolonged exchange intervals may be a better choice for pediatric chronic pancreatitis. Our study only compared stent indwelling durations of more than 6 months and 6 months or less. As asymptomatic patients tend to receive delayed stent exchange, this analysis may be subject to confounding by indication and survivor bias. Furthermore, the optimal replacement interval remains to be determined. Therefore, more prospective studies are warranted to address the above limitations in the future.

This study has several limitations inherent to its single-center, retrospective design, which precludes definitive causal inference and only allows for the identification of associations. First, the relatively limited sample size (*n* = 51) restricts the statistical power to detect subtler risk associations. Given the rarity of the disease and sample size, we adopted procedure-based analysis(*n* = 131). We also considered excluding repeated procedures and analyzing only the initial ERCP for each patient, yet the small number of patients and limited positive events led to unstable model outputs. Additionally, the single-center setting and the fact that procedures were performed by only two highly experienced endoscopists may further limit the generalizability of our findings to other centers with different operator expertise. Second, the lack of some available technical metrics, such as cannulation depth and procedure duration, precluded a comprehensive analysis of operator-dependent risk factors for PEP. Besides, several unresolved questions remain regarding: the optimal stent diameter threshold for pediatric ductal anatomy, optimal timing for stent exchange. An additional limitation pertains to the prophylactic use of non-steroidal anti-inflammatory drugs (NSAIDs). While NSAIDs were administered in 12 procedures within our cohort, they were not used in majority of cases, and relevant data of several patients could not be traced, reflecting the variability in clinical practice. Due to inconsistent and incomplete documentation regarding the timing, dosage, and specific type of NSAIDs administered for a substantial number of patients, we were unable to perform a reliable subgroup analysis to assess its potential impact on PEP incidence. This data limitation prevents us from fully evaluating the adherence to the ESGE guideline recommendations on routine NSAID use and its implications for our results. Therefore, dedicated studies focusing on pre-procedural NSAID administration in pediatric ERCP are urgently needed to support the development of specialized medication guidelines for children. Finally, the extended study period (spanning up to 10 years) introduces potential temporal bias, as diagnostic strategies and clinical practices may have evolved over time.

In conclusion, PBM and small stent diameter (≤ 5 Fr) are associated with increased PEP risk, although causality cannot be established. Extending stent exchange intervals beyond 6 months does not increase the risk of symptoms or acute pancreatitis. Individualizing stent replacement schedules to lower procedural burden appears feasible, which requires further validation in prospective studies.

## Data Availability

The datasets used and/or analyzed during the current study are available from the corresponding author on reasonable request.
